# *Thesium longiperianthium* (Santalaceae), a new replacement name for *T. brevibracteatum* P.C.Tam

**DOI:** 10.3897/BDJ.8.e59007

**Published:** 2020-11-06

**Authors:** Xuehong Xu, Wenjun Li, Khabibullo F Shomurodov, Ozodbek Abduraimov, Shukui Niu

**Affiliations:** 1 School of Ecology and Nature Conservation, Beijing Forestry University, Beijing, China School of Ecology and Nature Conservation, Beijing Forestry University Beijing China; 2 State Key Laboratory of Desert and Oasis Ecology, Xinjiang Institute of Ecology and Geography, Chinese Academy of Sciences, Urumqi, China State Key Laboratory of Desert and Oasis Ecology, Xinjiang Institute of Ecology and Geography, Chinese Academy of Sciences Urumqi China; 3 The Specimen Museum of Xinjiang Institute of Ecology and Geography, Chinese Academy of Sciences, Urumqi, China The Specimen Museum of Xinjiang Institute of Ecology and Geography, Chinese Academy of Sciences Urumqi China; 4 University of Chinese Academy of Sciences, Beijing, China University of Chinese Academy of Sciences Beijing China; 5 Institute of Botany, Academy of Sciences of the Republic of Uzbekistan, Tashkent, Uzbekistan Institute of Botany, Academy of Sciences of the Republic of Uzbekistan Tashkent Uzbekistan

**Keywords:** *Thesium brevibracteatum*, homonym, replacement name, Inner Mongolia

## Abstract

**Background:**

*Thesium brevibracteatum* P.C.Tam was described, based on the specimen *L.C.Chiu 5128* collected from Inner Mongolia, China. The name *Thesium brevibracteatum* Sumnev. is validly published and described for the type (*Korotkova E. E. et Titov V. S. 1502*) collected from Uzbekistan. *T. brevibracteatum* P.C.Tam is a later homonym of *T. brevibracteatum* Sumnev.

**New information:**

We propose *T. longiperianthium* as the replacemen name for *T. brevibracteatum* P.C. Tam.

## Introduction

*Thesium brevibracteatum* P.C.Tam was described, based on the specimen *L.C.Chiu 5128* collected from Inner Mongolia, China. It is a sub-fruticose herb up to 30 cm tall and grows on the sunny side of dunes and hills and steppes (Tam 1981, Tam 1988, Xia and Gilbert 2003, Zhao et al. 2020). In the protologue, the author indicated that *T. brevibracteatum* is similar to *T. longifolium* Turcz.(Sumnevich 1940), but is distinguished from the latter by the short bracts and long persistent perianth. As an endemic species, this species is distributed in Xilin Gol Meng, Horqin Right Front Banner and Hulun Buir, Inner Mongolia, China (Imzab 1990, Zhao et al. 2020).

The name *Thesium brevibracteatum* Sumnev. is validly published and described for the type (*Korotkova E. E. et Titov V. S. 1502*) collected from Uzbekistan (Sumnevich 1940). This species is a perennial soft-stemmed herb up to 25 cm tall. As indicated in the protologue, this species is close to *T. ramosissimum* Bobrov (Bobrov 1936) and *T. ramosum* Hayne (Hayne 1800). It differs from the first species by a very short peduncle - about 1 mm long (not 2-3 mm long), smaller nuts, non-woody roots, low and poorly-branched stems in the inflorescence area (only up to 60 cm and branched from the base) and from *T. ramosum* in its thin, woody roots and stems, branched in the inflorescence area, leaves with a single vein and shorter lateral bracts equal to half the length of the flower. As noted by Goloskokov (Goloskokov 1960), *T. brevibracteatum* Sumnev. is probably a southern, ecologically-isolated race of *T. ramosum* Hayne. The common distribution of the *T. brevibracteatum* Sumnev. is Central Asia (Western Tian Shan). It grows on the northern slopes in the upper parts of mountains on fine-grained slopes in woody and shrubby thickets (Sumnevich 1953). In addition to the type locality (Tashkent Alatau), this species was found in the Karatau mountains, Western Tian Shan (Kazakhstan) (Goloskokov 1960).

**Figure 1.**
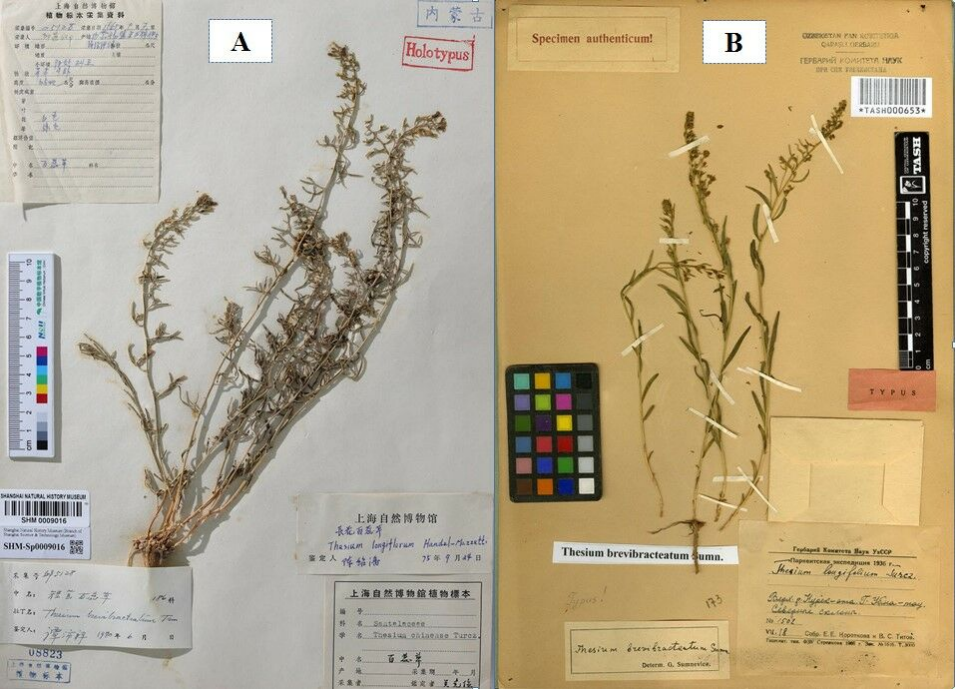
The holotype of *Thesium brevibracteatum* P.C.Tam (**A**) and *T. brevibracteatum* Sumnev. (**B**).

During the preparation of the checklist of vascular plants of Central Asia, we realised that the name *T. brevibracteatum* P.C.Tam is a later homonym of *T. brevibracteatum* Sumnev. (Art 53.1 of ICN, Turland et al. 2018). After checking the protologue and type specimens (Fig. 1), we determined that those two species are very different in the long peduncle and nut and the species *T. brevibracteatum* Sumnev. show shorter peduncle and smaller nut. This species is also different from some other species in neighbouring countries (Gubanov 1996; Krasnoborov and Malyshev 1992). In the protologue, the long persistent perianth is a key characteristic for *T. brevibracteatum* P.C.Tam. After checking the International Plant Name Index (https://www.ipni.org), the epithet “longiperianthium” has not previously been used for the genus *Thesium*. Thus, we propose the new name *T. longiperianthium* for *T. brevibracteatum* P.C.Tam.

## Taxon treatments

### Thesium longiperianthium

X.H.Xu & W.Jun Li nom. nov.

C7836306-6696-4795-8A3B-Eea9609Cd7A9

urn:lsid:ipni.org:names:77212687-1

≡ Thesium brevibracteatum P.C.Tam in P.C.Tam (1981) New materials on Santalaceae. Bulletin of Botanical Research 1: 73, nom. illeg.

#### Taxon discussion

*Thesium longiperianthium* X.H.Xu & W.Jun Li, nom. nov.≡ *Thesium brevibracteatum* P.C.Tam, 1981., New materials on Santalaceae. Bulletin of Botanical Research 1: 73. nom. illeg.

Type:—CHINA. Inner Mongolia: Xilin Gol Meng, Yikenao, 7 Sep 1965, *L.C.Chiu 5128* (HolotypeSHM 0009016!).

## Supplementary Material

XML Treatment for Thesium longiperianthium
